# Decellularized Swine Dental Pulp as a Bioscaffold for Pulp Regeneration

**DOI:** 10.1155/2017/9342714

**Published:** 2017-12-13

**Authors:** Lei Hu, Zhenhua Gao, Junji Xu, Zhao Zhu, Zhipeng Fan, Chunmei Zhang, Jinsong Wang, Songlin Wang

**Affiliations:** ^1^Molecular Laboratory for Gene Therapy and Tooth Regeneration, Beijing Key Laboratory of Tooth Regeneration and Function Reconstruction, School of Stomatology, Capital Medical University, Beijing 100050, China; ^2^Laboratory of Molecular Signaling and Stem Cells Therapy, Beijing Key Laboratory of Tooth Regeneration and Function Reconstruction, School of Stomatology, Capital Medical University, Beijing 100050, China; ^3^Department of Biochemistry and Molecular Biology, School of Basic Medical Sciences, Capital Medical University, Beijing 100069, China

## Abstract

Endodontic regeneration shows promise in treating dental pulp diseases; however, no suitable scaffolds exist for pulp regeneration. Acellular natural extracellular matrix (ECM) is a favorable scaffold for tissue regeneration since the anatomical structure and ECM of the natural tissues or organs are well-preserved. Xenogeneic ECM is superior to autologous or allogeneic ECM in tissue engineering for its unlimited resources. This study investigated the characteristics of decellularized dental pulp ECM from swine and evaluated whether it could mediate pulp regeneration. Dental pulps were acquired from the mandible anterior teeth of swine 12 months of age and decellularized with 10% sodium dodecyl sulfate (SDS) combined with Triton X-100. Pulp regeneration was conducted by seeding human dental pulp stem cells into decellularized pulp and transplanted subcutaneously into nude mice for 8 weeks. The decellularized pulp demonstrated preserved natural shape and structure without any cellular components. Histological analysis showed excellent ECM preservation and pulp-like tissue, and newly formed mineralized tissues were regenerated after being transplanted in vivo. In conclusion, decellularized swine dental pulp maintains ECM components favoring stem cell proliferation and differentiation, thus representing a suitable scaffold for improving clinical outcomes and functions of teeth with dental pulp diseases.

## 1. Introduction

Dental pulp is a soft tissue located in the pulp cavity. It is multistructural and composed of fibroblasts, odontoblasts, lymphocytes, endothelial cells, and other cells. Healthy dental pulp is important due to the formative, sensorial, and protective functions of the pulp-dentin complex. Its functions include nutrient supply to the teeth, dentin formation, sensing, defense, and other physiological functions. Pulpitis or pulp necrosis is a common disease caused by trauma and dental caries [1]. With the small root canal volume and narrow apical foramen, blood supply to the pulp is insufficient; therefore, once the pulp is inflamed, it often causes unrecoverable pulp necrosis.

With the development of regenerative medicine, regenerating pulp with biological functions is a new direction for treating dental pulp diseases. By transplanting stem cells along with growth factors and a biological scaffold into the prepared pulp cavity, stem cells can proliferate and differentiate into various cells in the pulp to achieve functional pulp regeneration [2]. Scaffolds play an important role in tissue engineering by providing a temporary, three-dimensional spatial structure, and extracellular matrix (ECM) component to maintain the regenerative environment and stem cell function [3, 4]. The ECM is essential for cell differentiation, proliferation, survival, and migration [5]. Scaffolds constructed to mimic ECM promote tissue and organ regeneration [6]. Various scaffolds have been used in dental pulp regeneration, including polylactic acid and collagen [7–11]; however, the major problem is lack of dental pulp extracellular matrix (ECM), thus limiting the ability to form dentin [12]. Therefore, it is essential to construct a dental pulp regeneration scaffold containing dental pulp ECM to promote odontoblast differentiation and dentin formation.

Acellular natural ECM is used as biological scaffold for preserving the anatomical structure of the natural organ and ECM. These include collagen I (COL I), collagen III (COL III), fibronectin, and laminin, which promote cell migration, proliferation, and differentiation [13]. Acellular natural ECM has been used for heart [6], liver [14], lung [15], and tissue regeneration. Currently, acellular ECM is mainly derived from animal tissue [16], and the xenogeneic ECM scaffold is mainly used for tissue regeneration [17]. Pigs share many anatomical and physiological characteristics with humans and act as donors for xenotransplantation. Porcine ECM has also been used for tissue regeneration in both preclinical and clinical studies [18, 19]. Swine have both deciduous and permanent dentitions (diphyodont), and their tooth structure is similar to that of humans. Whether swine dental pulp can be constructed as an acellular natural ECM scaffold for use in human pulp regeneration is unknown. Therefore, the present study investigated the structure and composition of decellularized swine dental pulp ECM and used swine acellular natural ECM combined with human dental pulp stem cells for pulp tissue regeneration.

## 2. Materials and Methods

### 2.1. Animals

Nine inbred miniature pigs (aged 12 months, weighing 45–50 kg) were purchased from the Animal Science Institute of Chinese Agriculture University in accordance with the Animal Care and Use Committee of Capital Medical University, Beijing, China. Balb/c nude mice were acquired from Vital River, Beijing, China. All animals were maintained under controlled temperature and light/dark cycles of 12 hrs each with food and water ad libitum. All animal experiments were performed in accordance with the Animal Welfare Act and approved by the Ethics Committee of Capital Medical University. Animals were acclimated to these conditions for 7 days before experiments and anesthetized by injecting ketamine chloride (6 mg/kg) and xylazine (0.6 mg/kg) intravenously before surgery.

### 2.2. Decellularization of Swine Dental Pulp

Dental pulps from eight swine were isolated for decellularization. Twelve-month-old swine were anesthetized by injecting ketamine chloride (6 mg/kg) and xylazine (0.6 mg/kg) intravenously. After anesthesia, four deciduous mandibular anterior teeth of each swine were extracted, exposing the permanent anterior teeth. The teeth were split by cutting along the long axis of teeth with scissors, which were extracted to acquire the dental pulp. The dental pulp was rinsed thoroughly in heparinized phosphate-buffered saline (PBS; Invitrogen, Carlsbad, CA, USA) for 15 min. Briefly, the pulp was placed in 10% sodium dodecyl sulfate (SDS; Sigma) in deionized water (w/v), continuously rocked in a shaker (MaxQ, Thermo Scientific, USA) for 32 h at 25°C and changed every 8 h. The residual SDS was removed by constant rocking in the shaker with sterile, deionized water at 25°C for 4 h. The samples were then placed in 1% Triton X-100 (Sigma) in deionized water (w/v), with continuous shaking at 25°C for 2 h, treated with 0.02 mg/ml DNase I (Sigma) and 20 mg/ml RNase (Sigma) for 1 h at 37°C, followed by heat inactivation at 75°C for 3 min. Finally, the decellularized dental pulp was washed with PBS for 2 h and placed in PBS containing 10 mg/ml streptomycin, 10,000 U/ml penicillin G, and 25 *μ*g/ml amphotericin B (Gibco, Carlsbad, CA, USA) for 12 h of sterilization.

### 2.3. Scanning Electron Microscopy Assay

Scanning electron microscopy (SEM) was used to investigate the microstructure of the decellularized dental pulp. The samples were fixed in 2.5% glutaraldehyde in 0.1 M of sodium cacodylate buffer (pH 7.2) at 4°C for 2 h. After 3 washes with the sodium dimethylarsinate buffer, the samples were postfixed in 1% osmium tetroxide in 0.1 M PBS for 1 h, dehydrated with gradient alcohol, and incubated with isoamyl acetate. After Au/Pd coating, EM images were taken using a Hitachi S-4800 SEM (Hitachi, Japan).

### 2.4. Human Dental Pulp Stem Cell (DPSC) Isolation and Culturing

DPSCs were isolated and cultured under good manufacturing practice (GMP) conditions as described in our previous studies [20]. Patients' teeth were acquired from Beijing Stomatological Hospital, Capital Medical University, and all treatments were performed per the approved guidelines. Informed consent was obtained from all patients. Briefly, six human impacted third molars (wisdom teeth) were collected from six healthy individuals (20–30 years old). The teeth were initially treated with 75% ethanol and washed with phosphate-buffered saline. The crown and root pulp tissue were obtained and digested with dispase II (4 mg/mL, Sigma-Aldrich) and collagenase type I (3 mg/mL, Sigma-Aldrich) for 1 h at 37°C. Single-cell suspensions were seeded into 10 cm culture dishes (BD Biosciences) and cultured in Eagle's medium with the alpha-modification (Invitrogen) with 15% fetal bovine serum (FBS, Gibco), 2 mM glutamine, 100 U/mL penicillin, and 100 mg/mL streptomycin (Invitrogen), and incubated in 5% carbon dioxide at 37°C.

### 2.5. Tooth Slice Preparation, Cell Seeding, and Transplantation into Immunodeficient Mice

Tooth slices were prepared and transplanted, per the previous study [7]. Twenty human impacted third molars (wisdom teeth) from twenty healthy individuals (20–30 years old) were acquired from Beijing Stomatological Hospital, Capital Medical University, per the approved guidelines and informed consent. Residual soft tissues around the teeth surface were removed with a scalpel, treated with 75% ethanol, and washed with phosphate-buffered saline (PBS). One-mm thick slices were prepared by transversally cutting at the cervical 1/3 of the root with a diamond-edged blade at low speed while cooling with sterile PBS. The pulp tissue was removed with forceps, and the predentin layer was removed with a handle Hedstrom file. Tooth slices were sterilized at 120°C for 20 min. The decellularized pulp was then sized to fit the lumen of the tooth slice. Tooth slices were divided into 2 groups: (1) DPSCs, acellular pulp and tooth slice and (2) control, empty tooth slice (no cells). Approximately 1*∗*10^6^ cells/ml of human DPSCs were injected into the acellular pulp by a 1-ml syringe and transplanted per the method of a previous study. After 2 months, tooth slices were acquired. Tooth slices were harvested from 3 healthy third molars containing the native dental pulp to serve as a control. All samples were fixed in 10% formalin at 4°C for 24 hours, demineralized with 10% formic acid at 4°C, and processed for histological analyses.

### 2.6. Histological and Immunostaining Analyses

Samples were fixed in 10% formalin and embedded in paraffin, and 5-*μ*m sections were prepared. Sections were stained with hematoxylin and eosin (H&E) for structural analyses. For fluorescent immunohistochemistry of collagen IV, laminin, fibronectin, integrin *β*1, and vimentin, deparaffinized slides were subjected to antigen retrieval in boiled sodium citrate buffer solution (pH 6.0) for 10 min and then cooled to room temperature for 1 h. Slides were blocked for 20 min in blocking buffer (5% bovine serum albumin and 0.01% Triton X-100 in PBS; Sigma). After blocking, rabbit polyclonal anti-collagen IV (Abcam, ab6586, 1 : 200), rabbit polyclonal anti-fibronectin (Abcam, ab2413, 1 : 200), rabbit polyclonal anti-laminin (Abcam, ab30320, 1 : 300), rabbit polyclonal anti-integrin *β*1 (Abcam, ab183666, 1 : 300), and rabbit polyclonal anti-vimentin (Santa Cruz, sc5565, 1 : 200) were incubated to attach overnight at 4°C. After incubation, slides were washed in PBS for 5 min. Alexa Fluor 594 goat anti-rabbit IgG (Life Technologies, USA, 1 : 500) and Alexa Fluor 488 goat anti-rabbit IgG (Life Technologies, USA, 1 : 500) were used to detect primary antibodies by incubating for 1 h at room temperature and then counterstaining the nuclei with DAPI (Invitrogen). DSPP expression was evaluated by immunohistochemistry. In brief, sections were treated with antigen retrieval as described above and incubated with mouse monoclonal anti-DSPP (Santa Cruz, sc73632, 1 : 200). Mouse-IgG*κ* binding protein-HRP (Santa Cruz, sc516102, 1 : 500) was added, and a DAB kit was used to detect primary antibodies. H&E stained images were captured using a Leica DM 4000 microscope (Leica, Germany), and immunofluorescent images were captured using a Leica TCS SP5 confocal microscope (Leica).

### 2.7. Statistical Analysis

All statistical calculations were performed using SPSS 13.0 statistical software. One-way ANOVA was used to determine statistical significance. *P* ≤ 0.05 was considered statistically significant.

## 3. Results

### 3.1. Morphological Observation of Decellularized Dental Pulp

**Figure 1:**
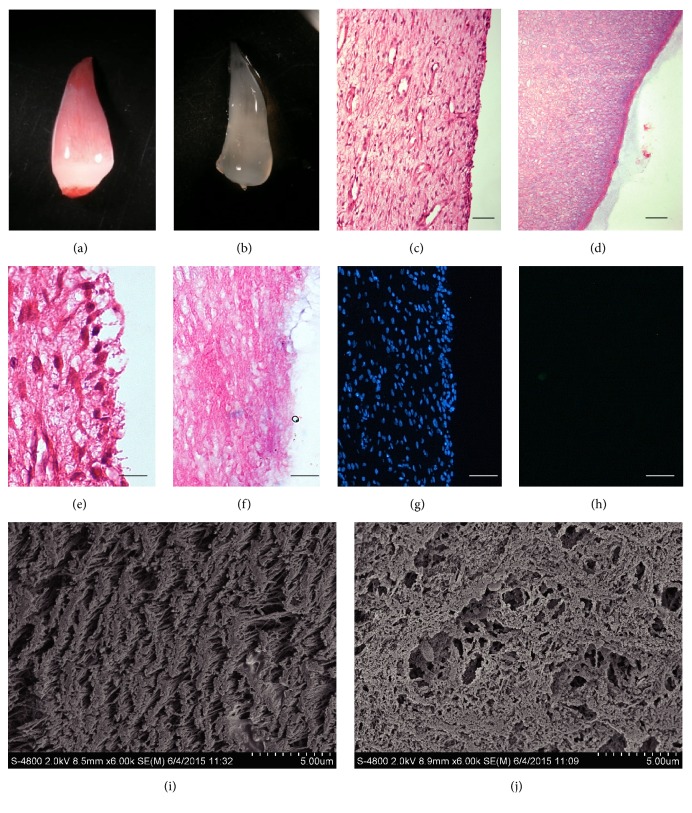
Images of swine dental pulp undergoing decellularization. At 0 h (a) and 32 h (b), there was a visible change from a normal pink to a mostly white, translucent appearance. H&E staining showed the native structures of pulp were well-preserved (c, d), while no cellular components were observed after 32 h of decellularization (e–h). SEM analyses showed the decellularization pulp present porose structure in transverse section (i) and vertical section (j). *n* = 4, scale bars: 100 *μ*m (c, d), 50 *μ*m (e, f), and 100 *μ*m (i, j).

After decellularization, the dental pulp gradually changed from pink to white-translucent, maintaining the gross shape of natural pulp (Figures 1(a) and 1(b)). Both H&E and 4′,6-diamidino-2-phenylindole (DAPI) analysis revealed that the cellular components of the dental pulp were completely removed, while the structure of the extracellular matrix (mainly collagen) was preserved (Figures 1(c)–1(h)). SEM images confirmed that the acellular and ECM structures with porous fibrous collagen were present in the decellularized dental pulp (Figures 1(i) and 1(j)).

### 3.2. Decellularized Pulp Extracellular Matrix Characterization

**Figure 2:**
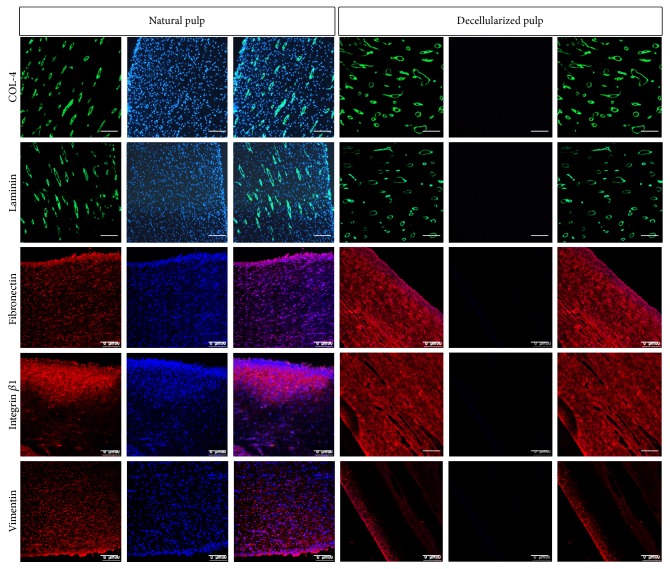
Characterization of the ECM components of decellularized pulp. Immunofluorescent staining showed that ECM proteins, Col-IV, laminin, fibronectin, intergrin *β*1, and vimentin were retained in the decellularized pulp, which was similar to the native pulp, except vimentin, which was decreased in decellularized pulp. Sections were counterstained with DAPI (blue). *n* = 4, scale bars: 100 *μ*m.

To investigate the extracellular matrix components of the decellularized dental pulp, five extracellular matrix proteins, collagen IV (COL-IV), laminin, fibronectin, integrin *β*1, and vimentin, were analyzed by immunofluorescence staining. The extracellular matrix of the decellularized dental pulp was well-preserved compared to the natural pulp. Collagen IV and laminin were mainly expressed in the basal membrane of the vascular-like structure. Fibronectin and integrin *β*1 were widely distributed. However, vimentin expression decreased after decellularization and was retained only in the odontoblast region (Figure 2).

### 3.3. Dental Pulp Regeneration Mediated by the Decellularized Pulp Scaffold

**Figure 3:**
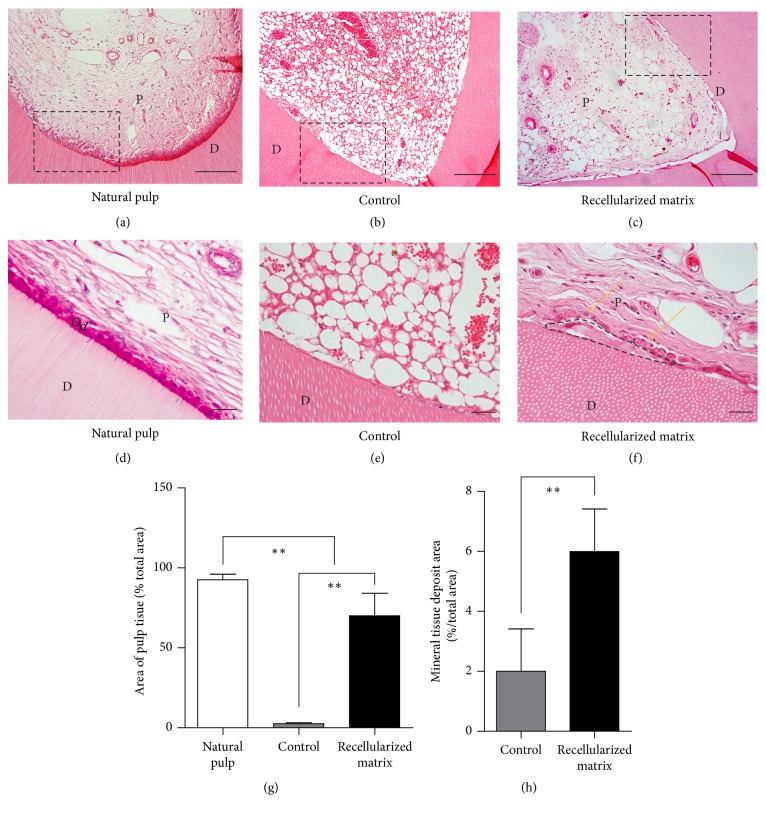
Cell reseeding and regeneration of dental pulp with decellularized pulp. Reseeded samples were transplanted subcutaneously in immunodeficient mice for 2 months, (a–c) H&E staining showed that pulp-like tissues were regenerated in recellularized matrix group, compared with natural pulp and blank control. (d–f) H&E staining showed the functional pulp was regenerated with a layer of mineral tissue regenerated (dashed line) between the dental pulp-like tissue and dentin interface, as well as a layer of polygonal cells (yellow arrow). (g) Quantitive analysis of pulp-like tissue regeneration area in control and recellularized matrix group. (h) Quantitive analysis of mineral tissue deposition area in natural pulp, blank control, and acellular pulp group. Values are mean ± SD, *n* = 10. One-way ANOVA was used to determine statistical significance, ^*∗∗*^*P* ≤ 0.01. D: dentin; P: pulp; Od: odontoblasts; scale bars: 50 *μ*m.

To evaluate the decellularized pulp matrix material for dental pulp regeneration, 1-mm thick human molar root slices were prepared, loaded with acellular pulp combined with DPSCs, and transplanted subcutaneously into nude mice for 2 months. H&E staining showed that the pulp-like tissues were regenerated in the recellularized matrix group, which was rich in fibrous tissues, accounting for 70% of the total area, while no fibrous tissues were generated in the blank group. Vascular-like structures were also found in the regenerated pulp-like tissue (Figures 3(a)–3(c) and 3(g)).

### 3.4. Immunohistochemistry Analysis of Regenerated Dental Pulp-Like Tissue

**Figure 4:**
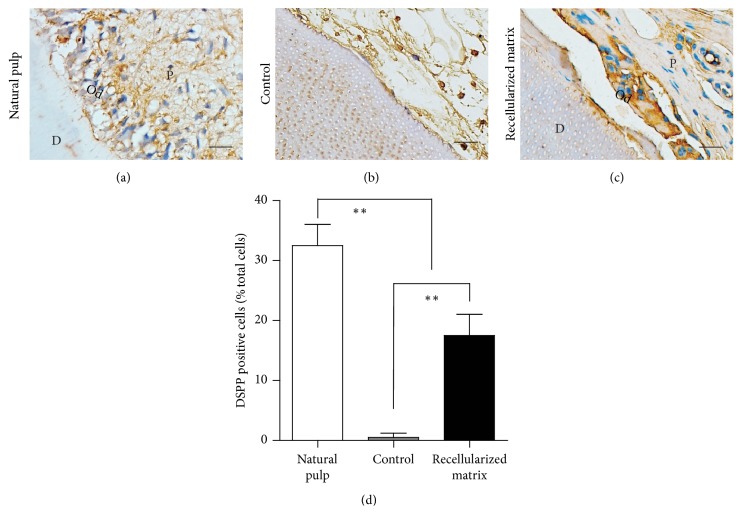
Immunohistochemistry analysis of regenerated pulp tissue. (a–c) Immunohistochemistry analysis showed polygonal cells layer highly expressing DSPP, which is a marker of odontoblast-like cells; no odontoblast, and mineralized tissue formation could be found in the control group. (d) Quantitive analysis of DSPP positive cells in natural pulp, control, and recellularized matrix group. Values are mean ± SD, *n* = 10. One-way ANOVA was used to determine statistical significance, ^*∗∗*^*P* ≤ 0.01. Scale bars: 100 *μ*m. D: dentin; P: pulp; Od: odontoblasts; scale bars: 50 *μ*m.

Functional regeneration is important for dental pulp regeneration. Dentin formation is one of the dental pulp's biofunctions; thus, the dentin interface, odontoblast differentiation, and dentin formation were analyzed. H&E staining showed that a functional pulp-like tissue was regenerated with a layer of newly formed, mineralized tissue in the recellularized group, lined by a layer of polygonal cells resembling odontoblast-like cells. The area of mineral tissue deposit was approximately 6% of the total area, which was significantly larger than that of natural pulp and of the control group (Figures 3(d)–3(f) and 3(h)), suggesting the regenerated pulp functions as a mineral tissue deposit. Immunohistochemical analysis showed that these cells are highly expressed DSPP, which is a marker of odontoblast cells. However, no odontoblasts or mineralized tissue formation was found in the control group (Figure 4). These results suggest that decellularized pulp can be used as a matrix material scaffold to promote typical dental pulp regeneration.

## 4. Discussion

In the present study, swine decellularized dental pulp was prepared as a porous scaffold. This procedure effectively removed cellular components while preserving the extracellular matrix proteins and natural structures. When combined with human DPSCs and transplanted into nude mice, the acellular pulp scaffold promoted the growth of dental pulp-like tissue and newly formed mineralized tissues. These results indicate that xenogeneic natural dental pulp ECM scaffolds can be used as suitable scaffolds for dental pulp regeneration.

Since Mooney first introduced tissue engineering-based dental pulp regeneration [21], many dental pulp regeneration studies have been conducted in various animal models. Stem cells, such as DPSCs, SHEDs, and SCAPs, combined with a scaffold can regenerate dentin-pulp tissues [7, 8, 22, 23]. Several scaffolds have been used to regenerate pulp; for example, beta-tricalcium phosphate (*β*-TCP) combined with dental pulp stem cells from porcine primary teeth successfully mediated the regeneration of dentin-pulp tissues in miniature pigs [24]. Platelet-rich plasma (PRP) combined with dental pulp cells (DPC) promotes dental pulp regeneration in small dog canals [25]. However, predictability of the outcomes and organization of the newly formed tissues are unsatisfactory due to the unavailability of a suitable scaffold for pulp regeneration.

A suitable scaffold is necessary for functional dental pulp regeneration. With its multiple cell types and structures, it is impossible to functionally regenerate dental pulp with a single scaffold. Synthetic composite scaffolds have a complex construction process, lack ECM components, and have an unsatisfactory effect on tissue regeneration. The native ECM is crucial in organogenesis and homeostasis, by mediating biophysical stimuli, biochemical and molecular signals, and spatial organization. Compared with other scaffolds, acellular tissue and organs are relatively easier to construct as composite scaffolds, which maintain the natural structure and extracellular matrix components, and have been used to regenerate various tissues and organs with favorable results [26].

Previous studies have verified that human dental pulp from healthy extracted teeth can be decellularized to form an ECM scaffold. The results presented an effective method for decellularizing human pulp, which was used by a previous study [17], and the decellularized ECM supported the proliferation and differentiation of SCAP [27]. The results showed decellularized pulp scaffolds can promote DPSCs to differentiate into odontoblast-like cells near the dentinal walls, indicating decellularized pulp ECM may be a promising scaffold for pulp regeneration. However, with the limited sources and small size of human pulp, allogeneic acellular pulp ECM cannot fulfill clinical applications. Xenogeneic acellular tissues have been used for tissue regeneration and rarely cause immune rejection, as the extracellular matrix proteins are well-conserved [28]. Swine are widely used in biomedical studies [29], including dental pulp regeneration, since the structure of swine dentition is similar to that of humans [30]. Thus, it was necessary to evaluate whether swine dental pulp can be prepared as an acellular scaffold for dental pulp regeneration.

In the present study, we did not use the decellularization method of previous studies [17], with hypertonic buffer washed for 48 hours, followed by 3 cycles of 1% SDS for 24 h and 1% Triton X-100 for 24 h. The pulp was decellularized together with the tooth slice, without being extracted from the root canal. Swine dental pulp is larger than human pulp and thus unsuitable for this decellularization process. We generated a swine acellular pulp scaffold after root canal extraction, by using our previous decellularization method used on rat submandibular glands [31]. The results showed the gross morphology of swine dental pulp was well-preserved after decellularization treatment. No cells were retained in the dental pulp after decellularization, and the acellular pulp presented porous structure, containing the extracellular matrix proteins, Col-IV, laminin, fibronectin, integrin *β*1, and vimentin. However, vimentin expression was decreased and retained only in the odontoblast layer. Vimentin belongs to the intermediate filaments, makes up the cytoskeleton, is expressed in the preodontoblasts, and modulates odontoblast differentiation [32]; thus, the decreased vimentin in the acellular pulp was due to decellularizing of the preodontoblasts.

Natural ECM proteins play crucial roles in organ development [33–38], tissue repair, and regeneration [39]. In the present study, different matrix proteins have specific expression patterns. Col-IV and laminin were expressed in vascular-derived basement membranes and involved in blood vessel formation [40, 41]. The expression of Col-IV and laminin was concentrated in the vascular-like basement membrane region, suggesting that these two proteins may participate in the pulp vascular regeneration. Fibronectin and integrin *β*1 were widely expressed, suggesting that these two proteins were involved in maintaining the pulp's gross structure. Vimentin was mainly expressed in the odontoblast layer and may be related to odontoblast differentiation and dentin formation. The site-specific expression of extracellular matrix proteins can entitle the acellular pulp as a natural scaffold with a complex structure. Human DPSCs combined with the swine acellular dental pulp scaffold were transplanted subcutaneously into nude mice to observe dental pulp regeneration. Swine acellular pulp regenerated pulp-like tissues with rich vascularity and generated a layer of newly formed mineralized tissues in the pulp-dentin interface, suggesting functional pulp regeneration was achieved. This effect may be due to its complex structure and different ECM component characteristics, thus recruiting cells to different sites and differentiating into multiple cell types to promote dental pulp regeneration.

Here, we showed 10% sodium dodecyl sulfate (SDS) combined with TritonX-100 can conveniently and effectively decellularize swine pulp. The decellularized ECM allowed for pulp-like tissue formation after ectopic subcutaneous implantation in immunocompromised mice. This was the first verification that decellularized swine pulp can mediate human DPSC-based pulp regeneration. These data can simplify the process and broaden the potential clinical applications of acellular pulp ECM for pulp regeneration.

## 5. Conclusions

In conclusion, swine dental pulp, after being decellularized, can be prepared as a porous scaffold. This scaffold preserved the extracellular matrix proteins and natural structures and could promote stem cell-mediated pulp regeneration in vivo. Thus, dental pulp extracellular matrix scaffolds can be used as suitable scaffolds for dental pulp regeneration.
